# Estimation of the Basic Reproductive Ratio for Dengue Fever at the Take-Off Period of Dengue Infection

**DOI:** 10.1155/2015/206131

**Published:** 2015-08-25

**Authors:** Jafaruddin, Sapto W. Indratno, Nuning Nuraini, Asep K. Supriatna, Edy Soewono

**Affiliations:** ^1^Departemen Matematika, FMIPA, Institut Teknologi Bandung, Bandung, Indonesia; ^2^Jurusan Matematika, FST, Universitas Nusa Cendana, Kupang, Indonesia; ^3^Jurusan Matematika, FMIPA, Universitas Padjadjaran, Bandung, Indonesia

## Abstract

Estimating the basic reproductive ratio *ℛ*_0_ of dengue fever has continued to be an ever-increasing challenge among epidemiologists. In this paper we propose two different constructions to estimate *ℛ*_0_ which is derived from a dynamical system of host-vector dengue transmission model. The construction is based on the original assumption that in the early states of an epidemic the infected human compartment increases exponentially at the same rate as the infected mosquito compartment (previous work). In the first proposed construction, we modify previous works by assuming that the rates of infection for mosquito and human compartments might be different. In the second construction, we add an improvement by including more realistic conditions in which the dynamics of an infected human compartments are intervened by the dynamics of an infected mosquito compartment, and vice versa. We apply our construction to the real dengue epidemic data from SB Hospital, Bandung, Indonesia, during the period of outbreak Nov. 25, 2008–Dec. 2012. We also propose two scenarios to determine the take-off rate of infection at the beginning of a dengue epidemic for construction of the estimates of *ℛ*_0_: scenario I from equation of new cases of dengue with respect to time (daily) and scenario II from equation of new cases of dengue with respect to cumulative number of new cases of dengue. The results show that our first construction of *ℛ*_0_ accommodates the take-off rate differences between mosquitoes and humans. Our second construction of the *ℛ*_0_ estimation takes into account the presence of infective mosquitoes in the early growth rate of infective humans and vice versa. We conclude that the second approach is more realistic, compared with our first approach and the previous work.

## 1. Introduction

Dengue is a mosquito-borne viral disease found in more than 100 countries around the world which are mostly located in tropical and subtropical countries. This disease is transmitted through the bites of female* Aedes aegypti*. In recent years, dengue transmission has increased predominantly in urban and semiurban areas and has become a major international public health concern. Controlling of dengue fever has been conducted continuously, but the spread of dengue virus is still increasing in many countries. Many efforts have been conducted to control the spread of the virus, for instance, a reduction in the population of* Aedes aegypti* in the field [1]. Fumigation methods have been used to reduce mosquitoes, and the use of temephos has been utilized to reduce the larvae. Until recently, there was no vaccine against any of the four virus serotypes (DEN-1, DEN-2, DEN-3, and DEN-4). To cure patients, treatment in hospitals is usually given in the form of supportive care, which includes bed rest, antipyretics, and analgesics [2]. Mathematical models have proved to be useful tools in the understanding of dengue transmission [3–5]. So far the dynamics of the dengue transmission are still an interesting issue in epidemiological modeling.

The first model of dengue transmission and the stability analysis of equilibrium points are shown in [6] in which the basic reproductive ratio *ℛ*_0_ is constructed from the stability condition of the disease-free equilibrium. The general concept of *ℛ*_0_ can be seen in [7, 8] which was adapted to various infectious disease models [9, 10]. The difficulty in using *ℛ*_0_ for measuring the level of dengue endemicity in the field is that *ℛ*_0_ often depends on several parameters (biological, environmental, transmission, etc), which are difficult to be obtained from the field. With limited information about mosquitos such as the mosquito population size the estimate of *ℛ*_0_ can not be computed from the dynamical model only. With daily human incidence being the only available data, it is natural to ask how to extract relevant parameters from the data to fit in the model. A simple approach has been achieved by assuming that both a human and mosquito undergo linear growth at the early state of infection. As shown in [11–14] the estimation does not distinguish between the growth rate of an infected human and the growth rate of infected mosquitoes and does not yet consider how the interaction between infected mosquitoes and infected humans influences the early growth of a dengue epidemic. There are other problems that are treated in a recent paper [15] which examines two models vector-borne infections, namely, dengue transmission. In this paper, we construct *ℛ*_0_ estimation by taking into account the difference between the growth rate of an infected human and infected mosquito and the effect of the interaction between them and the characteristics of dengue incidence data.

The existing data do not specify the infection status (age of infection) of each patient. Hence we assume that people who come to the hospital should be identified as dengue patient at the end of incubation period (approximately at the 7th day after contact with infected mosquito). Also, we assume that there are no available alternative hosts as blood sources and there are no death and recovery in the early days of dengue infection. The main purpose of this paper is to construct *ℛ*_0_ estimation of the real conditions in the field, in which only the daily incidence data are available. Based on the dengue transmission model in [6], we build two different constructions for *ℛ*_0_ estimation and used them for estimating the value of *ℛ*_0_ for dengue incidence data between the dates Nov. 25, 2008, and 2012 from SB Hospital, Indonesia.

The organization of the paper is given as follows. In Section 2 a dynamical system of a host-vector transmission model is introduced. Based on this dynamical system we derive the basic reproductive ratio of the model. The constructions of the proposed *ℛ*_0_ estimation are presented in Section 3. Formulations of the take-off rate of dengue infection are presented in Section 4. We apply the formula to the real data of dengue incidence from SB Hospital, Bandung, Indonesia. All the numerical results are given in Section 5 along with the insights and interpretation from the numerical results. We provide conclusions in Section 6.

## 2. Basic Reproductive Ratio of the Dengue Transmission Model

**Figure 1:**
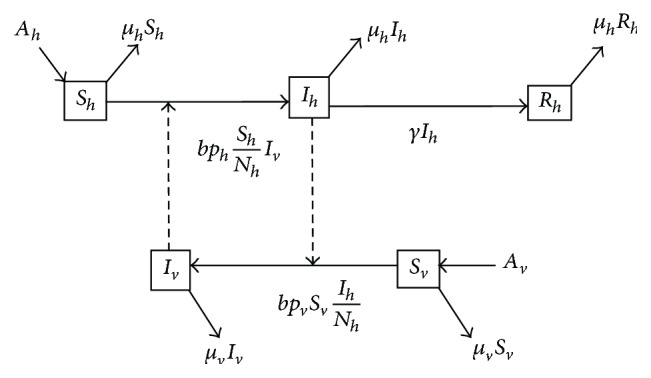
The diagram of dengue transmission model.

Generally, for a vector-borne disease, *ℛ*_0_ was understood as the number of persons who would be infected from a single person initially infected by a mosquito [3, 16]. In the host-vector system, the basic reproductive ratio *ℛ*_0_ is defined as the expected number of secondary infections resulting directly from a single infected individual, in a virgin population, during the infection period [7]. The general host-vector dengue transmission model was conducted in [6]. Here, we assume that there are no alternative hosts available, as blood sources *m* = 0 for system in [6]. Modification of the dengue transmission model in [6], is schematically represented by the diagram in Figure 1, where

 
*A*_*h*_: recruitment rate of host population;
 
*N*_*h*_: number of host populations;
 
*S*_*h*_: susceptible host population size;
 
*I*_*h*_: infected host population size;
 
*R*_*h*_: recovered/immunes host population size;
 
*p*_*h*_: successful transmission probability within host population;
 
*μ*_*h*_: birth/death rate of host population;
 
*γ*: recovery rate of host population;
 
*A*_*v*_: recruitment rate of vector population;
 
*N*_*v*_: number of vector populations;
 
*S*_*v*_: susceptible vector population size;
 
*I*_*v*_: infected vector population size;
 
*p*_*v*_: transmission probability within the vector population;
 
*μ*_*v*_: death rate of vector population;
 
*b*: biting rate of vector population.

The corresponding model of dengue transmission in the short period dengue infection is given in [17] as follows:

(1) dShdt=Ah−bphShIvNh−μhSh,dIhdt=bphShIvNh−γIh−μhIh,dRhdt=γIh−μhRh,dSvdt=Av−bpvSvIhNh−μvSv,dIvdt=bpvSvIhNh−μvIv.

Here, we give a short explanation for system (1) as follows. The effective contact rate to human *bp*_*h*_ is the average number of contacts per day that would result in infection, if the vector is infectious. Furthermore, the effective contact rate *bp*_*v*_ defines the average number of contacts per day that effectively transmit the infection to vectors.

First of all, we determine equilibrium points for the system (1). Because *N*_*h*_ and *N*_*v*_ are assumed to be constant then from system (1) ones obtain *S*_*h*_ + *I*_*h*_ + *R*_*h*_ = *N*_*h*_ = *A*_*h*_/*μ*_*h*_ and *S*_*v*_ + *I*_*v*_ = *N*_*v*_ = *A*_*v*_/*μ*_*v*_. Consequently, we obtain a disease-free equilibrium point of system (1), which is given by

(2) $$\begin{array}{c} E_{0}=\left(\;S_{h0}=\frac{A_{h}}{\mu_{h}},I_{h0}=0,R_{h0}=0,S_{v0}=\frac{A_{v}}{\mu_{v}},I_{v0}=0\;\right), \end{array}$$

and the endemic equilibrium point in the form of

(3) $$\begin{array}{c} E_{1}=\left(\;S_{h}^{*},I_{h}^{*},R_{h}^{*},S_{v}^{*},I_{v}^{*}\;\right), \end{array}$$

where

(4) Sh∗=μvγ+μhNh2+μvNhNv2phμhNh+bphNv,Ih∗=μvμhNhb2phpv/μvγ+μhNv/Nh−1bpvμh+bphρ,Rh∗=μvNhγb2phpv/μvγ+μhNv/Nh−1bpvμh+bphρ,Sv∗=phμhNh+bpvphNvbph1+bpvμh,Iv∗=μvμhNhγ+μhb2phpv/μvγ+μhNv/Nh−1bphμvγ+μh+μhbpv.

Next, we derive the basic reproductive ratio *ℛ*_0_. *ℛ*_0_ is often conveniently found using a next generation method [7, 8, 18, 19]. We obtain the next generation matrix of system (1), at *E*_0_, as follows

(5) $$\begin{array}{c} NGM=\left[\begin{array}{cc} 0 & \frac{bp_{h}}{\mu_{v}}\\ \frac{bp_{v}}{\left(\gamma +\mu_{h}\right)}\frac{N_{v}}{N_{h}} & 0 \end{array}\right]. \end{array}$$

Based on the definition in [20], NGM represents that the number of *bp*_*h*_/*μ*_*v*_ is the generation factor of dengue transmission, from mosquito to human. It means that one mosquito infects *bp*_*h*_ humans per unit of time during its infection period 1/*μ*_*v*_, and the number of (*bp*_*v*_/(*γ* + *μ*_*h*_))(*N*_*v*_/*N*_*h*_) is the generation factor of dengue transmission, from human to mosquito. This represents that one human infects *bp*_*v*_(*N*_*v*_/*N*_*h*_) mosquitoes during one's infection period 1/(*μ*_*h*_ + *γ*). NGM in (5) have two eigen values, that are $\pm \sqrt{(b^{2}p_{h}p_{v}/\mu_{v}\left(\gamma +\mu_{h}\right))(N_{v}/N_{h})}$. Therefore the basic reproductive ratio of system (1) is the largest eigen value of NGM, that is

(6) $$\begin{array}{c} R_{0}=\sqrt{\frac{b^{2}p_{h}p_{v}}{\mu_{v}\left(\gamma +\mu_{h}\right)}\frac{N_{v}}{N_{h}}}. \end{array}$$

It is clear that the endemic equilibrium *E*_1_ in (3) exists if *ℛ*_0_ > 1. In the next section we propose two constructions of an *ℛ*_0_ estimation, at the initial growth of dengue infection, based on incidence data.

## 3. Construction for *ℛ*_0_ Estimation

Predicting a basic reproductive ratio is not easily done in the field. In the real-life situation, information about the number of mosquitoes, precisely the ratio between mosquito and human population, is limited. Based on the incidence data, we will estimate some related parameters, for the construction of the basic reproductive ratio *ℛ*_0_, from dengue incidence data.

This construction is motivated by previous work in [12], which assumes that the infective growth rate is linear at the early state of infection. The exponential growth of the infective compartment, at the early state of infection, is commonly found in the estimation of the basic reproductive ratio. All estimation of *ℛ*_0_, in previous works [11, 12, 14, 21, 22], have been conducted based on the assumption that *K*(*t*) ∝ *e* ^*λt*^ varies, where *K*(*t*) is the cumulative number of dengue cases, and *λ* is called the force of infection [12, 14]. Chowell et al. [11] called *λ* the initial growth rate of dengue epidemic. Here, we will call *λ* the take-off rate of dengue infection. In the following section, we build two constructions to estimate *ℛ*_0_ in (6), which is derived from a dynamical system of the host-vector dengue transmission model in system (1).

First of all we assume the construction of *ℛ*_0_ estimation from equation (3) in [12]. The construction of *ℛ*_0_ estimation at *E*_0_ is based on the assumptions that the numbers of infected human population *I*_*h*_ and infected mosquito population *I*_*v*_ grow exponentially at the same rate at the short time period relative to each other:

(7) Iht≈Ih0eλt,Ivt≈Iv0eλt,

where *I*_*h*0_ and *I*_*v*0_ are constant and *λ* is the take-off rate of the initial growth dengue epidemic. Moreover, the number of nonsusceptible hosts and vectors can be assumed to be negligible and by assuming (7) is “*like solution*” of linearized system (1) for *ℛ*_0_ > 1. These assumptions are also used in [11, 14] for calculating the force of infection from the spatial data of a dengue epidemic. Next, by substituting (7) with (1), and assuming that *S*_*h*_ ~ *N*_*h*_ and *V*_*v*_ ~ *N*_*V*_ at the early state of an epidemic, we get

(8) λγ+μh+1Ih0=bphγ+μhIv0,

(9) λμv+1Iv0=bpvμvNvNhIh0.

Multiplying (8) and (9), and using (6), one gets the construction of the *ℛ*_0_ estimation as follows:

(10) R0F2λμh+γ+1λμv+1=b2phpvμvμh+γNvNh=R02,

which is equivalent to

(11) $$\begin{array}{c} R_{0F}=\sqrt{\frac{\lambda^{2}}{\mu_{v}\left(\mu_{h}+\gamma \right)}+\frac{\left(\mu_{v}+\mu_{h}+\gamma \right)\lambda }{\mu_{v}\left(\mu_{h}+\gamma \right)}+1}. \end{array}$$

The value of *ℛ*_0*F*_ in (11) is used to estimate *ℛ*_0_ in (6), where *λ* is estimated from the dengue incidence data. Note that since *λ* > 0, then *ℛ*_0*F*_ > 1. Note also that a small increase of the value *λ* in the interval 0 < *λ* < 1 causes a significant increase of the value *ℛ*_0*F*_.

Next we introduce our construction of *ℛ*_0_ estimation. The first construction is derived under the assumption that, at the beginning of dengue infection, the take-off rate of the host and vector varies differently; that is

(12) Iht≈Ih0eλt,Ivt≈Iv0ekλt,

where *k* > 0 is the rate of infected mosquitoes per human index. Using the same process as in the derivation of *ℛ*_0*F*_, one obtains

(13) R0MF2λμh+γ+1kλμv+1=b2phpvμvμh+γNvNh=R02

or, equivalently,

(14) $$\begin{array}{c} R_{0MF}=\sqrt{\frac{k\lambda^{2}}{\mu_{v}\left(\mu_{h}+\gamma \right)}+\frac{\left(\mu_{v}+k\left(\mu_{h}+\gamma \right)\right)\lambda }{\mu_{v}\left(\mu_{h}+\gamma \right)}+1}. \end{array}$$

Note that *ℛ*_0*F*_ is a special case from *ℛ*_0*MF*_; that is, if *k* = 1 then *ℛ*_0*F*_ = *ℛ*_0*MF*_. In reality *ℛ*_0*MF*_ is more realistic than *ℛ*_0*F*_. From (11) and (14) we obtain

(15) $$\begin{array}{c} \frac{R_{0MF}^{2}}{R_{0F}^{2}}=1+\frac{\left(k-1\right)\lambda }{\lambda +\mu_{v}}. \end{array}$$

**Figure 2:**
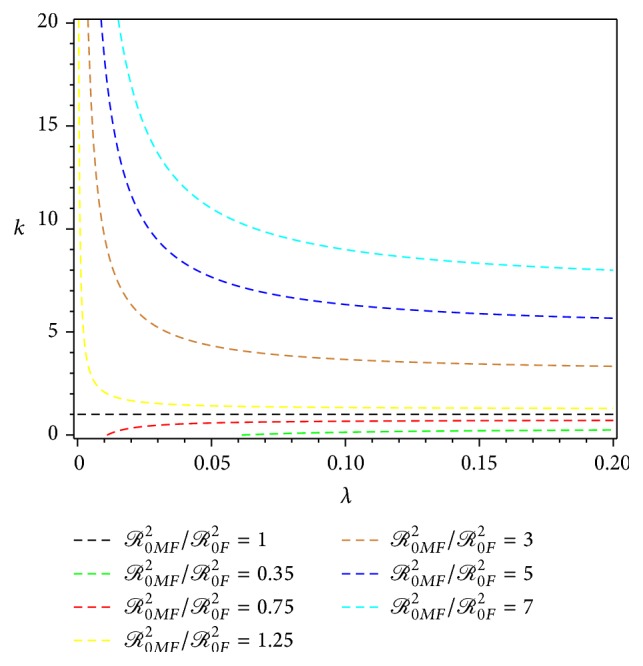
Level set for *ℛ*_0*MF*_^2^/*ℛ*_0*F*_^2^ in (15) for data set {*μ*_*v*_ = 1/30, *λ* ∈ [0,0.20], *k* ∈ [0,20]}.

The comparison between *ℛ*_0*MF*_ and *ℛ*_0*F*_ is shown as the level set of (15) in Figure 2.

Figure 2 shows that the variation in take-off rates might significantly change the values of *ℛ*_0*F*_ and *ℛ*_0*MF*_. Both estimations are derived under the assumption that the pure exponential growth phase, of an epidemic, increases or has a fluctuating increase. Accordingly the estimates of *ℛ*_0_, obtained from *ℛ*_0*F*_ and *ℛ*_0*MF*_, are affected by biasness; that is, the derivations of *ℛ*_0*F*_ and *ℛ*_0*MF*_ use the extreme assumption in the early dengue epidemic that the dynamics of an infected human are not yet intervened by the presence of an infected mosquito's influence, and vice versa.

The second construction of *ℛ*_0_ estimation is done in order to revise the first construction. We assume that the exponential growth of the infective host, at the early state of infection, is slightly affected by the growth of the infective vector, and vice versa. The main problem here is to find the best fit interval (here we use the terminology take-off period) where exponential growth occurs. Statistically, this situation can be related to the linear phase fit in a short time period.

We define the initial growth in a period of time as the initial value problem; that is,

(16) Iht=Iv0aeηt+be−ηt+Ih0ceηt+de−ηt,Ivt=Ih0peηt+qe−ηt+Iv0reηt+se−ηt,Ih0,Iv0=Ih0,Iv0,Ih′0,Iv′0=ηIv0,ηIh0.

We obtain

(17) a=12,b=12,c=12,d=12,p=12,q=12,r=12,s=12

such that the solution of (16) is obtained; namely,

(18) Iht=Ih0coshηt+Iv0sinhηt,Ivt=Iv0coshηt+Ih0sinhηt.

The main idea from (18) is to extract the relevant parameter(s) during the early state of infection, in which linear growth rate of infection is assumed to take place. Next we substitute (18) with the expression for the derivative of *I*_*h*_ and *I*_*v*_ in (1) by simple algebraic manipulation; we then obtain a homogeneous system of equations in cosh⁡(*ηt*) and sinh⁡(*ηt*), namely,

(19) $$\begin{array}{c} A\left[\begin{array}{c} cosh\left(\eta t\right)\\ sinh\left(\eta t\right) \end{array}\right]=\left[\begin{array}{c} 0\\ 0 \end{array}\right], \end{array}$$

where coefficient matrix is

( 20 ) A=Ih0η−bphIh0+γ+μhIv0Iv0η−bphIv0+γ+μhIh0Iv0η−bpvρIv0+μvIh0Ih0η−bpvρIh0+μvIv0.

A nontrivial solution from (19) exists if |*A* | = 0, which gives

(21) η2Nh−bpvNv+Nhbphη−μvγ+μhNh+b2pvNvph

as the coefficient of *I*_*h*0_^2^ and *I*_*v*0_^2^ must be equal to zero. Equivalently, from (21), we obtain

(22) R0A2−η2μvγ+μh+bph+pvNv/Nhμvγ+μhη+1=b2phpvμvμh+γNvNh=R02.

Therefore we have

(23) $$\begin{array}{c} R_{0A}=\sqrt{-\frac{\eta^{2}}{\mu_{v}\left(\gamma +\mu_{h}\right)}+\frac{b\left(p_{h}+p_{v}\rho \right)}{\mu_{v}\left(\gamma +\mu_{h}\right)}\eta +1}, \end{array}$$

where *ρ* = *N*_*v*_/*N*_*h*_ is the known mosquitoes per person index. We obtain (23), as a new construction for estimation *ℛ*_0_, as a function of *η*. The construction of *ℛ*_0_ in (23) is related to a short time interval where linear growth may still take place. Here, we will estimate the value of *η* during the early state of infection, in which a linear growth of infection is assumed to take place.

Note that *ℛ*_0*A*_ increases with respect to *η*, 0 ≤ *η* ≤ *b*(*p*_*h*_ + *p*_*v*_ *ρ*)/2. Based on the definition that *η* is probability, per unit of time, a susceptible becomes infected at the beginning of a dengue epidemic (see [7]), with assumption that *b*(*p*_*h*_ + *p*_*v*_ *ρ*)/2 < 1. This assumption means that the maximum take-off rate of dengue virus infection is defending on the index mosquito per human. This condition implies

(24) $$\begin{array}{c} \frac{2\eta -bp_{h}}{bp_{v}}\leq \rho \leq \frac{2-bp_{h}}{bp_{v}}. \end{array}$$

*ℛ*_0*A*_/*ℛ*_0*F*_ ratio in the early phase of dengue epidemics could be significantly larger or smaller than one depending on the value of *ρ*, in (24), and the value of *λ*. In Section 4, we determine *η* as a function of *λ* at the beginning of a dengue epidemic. We will further analyze the level set of the ratio *ℛ*_0*A*_/*ℛ*_0*F*_. Considering that the value of *ℛ*_0_ estimation depends on the value of take-off rate *λ*, we discuss a method to formulate the take-off rate in the following section.

## 4. Formulation of the Take-Off Rate

Here we derive two scenarios for the derivation of the take-off rate (t.o.r.) at the beginning of every take-off period (t.o.p.) of dengue infection. Both scenarios are based on the same assumptions, but with a slightly different implementation for real-life dengue epidemics. Therefore, the main problem here is to find the best fit interval (here we use the terminology “take-off period”) where exponential growth occurs.

The first scenario is done under the assumption that, during early infection, recovery and natural death do not yet occur. Note that the original data taken from the hospital is in the form of daily new cases. Let *I*(*t*) be the number of new cases of infection; we then have *I*(*t*) = *dI*_*h*_/*dt* (see in [14]). Therefore, from (7), it is

(25) $$\begin{array}{c} I\left(\;t\;\right)=\lambda I_{h0}e^{\lambda t},\;\text{with }I_{0}=I\left(0\right)=\lambda I_{h0} \end{array}$$

and, from (12), it is

(26) It=ηIv0cosh⁡ηt+Ih0sinh⁡ηt,with  I0=I0=ηIv0.

By using the Taylor expansion around *λ* = 0 for (25) and around *η* = 0 for (26), we obtain

(27) It≈λI0t+I0,It≈η2λI0t+I0,

respectively. Consequently, we have a relation that *λ* = *η*. Thus *η* at *ℛ*_0*A*_ in (23) can be replaced by *λ*. By comparing (11) and (23), we have

(28) $$\begin{array}{c} \frac{R_{0A}^{2}}{R_{0F}^{2}}=1+\frac{\lambda }{\lambda +\mu_{v}}\left(\;\frac{b\left(p_{h}+p_{v}\rho \right)-\lambda }{\lambda +\mu_{h}+\gamma }-1\;\right). \end{array}$$

Also *η* in (24) can be replaced by *λ* such that we obtain

(29) $$\begin{array}{c} \frac{2\lambda -bp_{h}}{bp_{v}}\leq \rho \leq \frac{2-bp_{h}}{bp_{v}}. \end{array}$$

The ratio *ℛ*_0*A*_^2^/*ℛ*_0*F*_^2^ ≥ 1, if and only if *ρ* ≥ (2*λ* + *μ*_*h*_ + *γ* − *bp*_*h*_)/*bp*_*v*_, and *ℛ*_0*A*_^2^/*ℛ*_0*F*_^2^ < 1, if and only if *ρ* < (2*λ* + *μ*_*h*_ + *γ* − *bp*_*h*_)/*bp*_*v*_. Because of (2*λ* − *bp*_*h*_)/*bp*_*v*_ < (2*λ* + *μ*_*h*_ + *γ* − *bp*_*h*_)/*bp*_*v*_, we have (2*λ* + *μ*_*h*_ + *γ* − *bp*_*h*_)/*bp*_*v*_, which belongs to the range of *ρ* for small *λ*.

The second scenario is conducted under the same assumption as the first scenario. But, here, we regress between the number of new cases in a day *I*(*t*) = *dI*_*h*_/*dt* and cumulative number of new cases *K*(*t*), where we have the initial value *K*(0) = *K*_0_ = *I*_0_ = *I*(0) = *λI*_*h*0_ from (7) and *K*(0) = *K*_0_ = *I*_0_ = *I*(0) = *ηI*_*v*0_ from (18). Therefore, by using integral equation *K*(*t*) − *K*(0) = ∫_0_^*t*^ *I*(*τ*)*dτ* with *I*(*τ*) from the first equation in (7) and *I*(*τ*) from the first equation in (18), we obtain

(30) It=λKt+1−λK0,It=η2Kt+1−η2K0,

respectively. It is clear that *η* ^2^ = *λ* so that *η* in *ℛ*_0*A*_ at (23) can be replaced by $\sqrt{\lambda }$. If (23) is divided by (11), we obtain

(31) $$\begin{array}{c} \frac{R_{0A}^{2}}{R_{0F}^{2}}=1+\frac{\sqrt{\lambda }}{\lambda +\mu_{v}}\left(\;\frac{b\left(p_{h}+p_{v}\rho \right)-\sqrt{\lambda }}{\lambda +\mu_{h}+\gamma }-\sqrt{\lambda }\;\right). \end{array}$$

Also *η* in (24) can be replaced by $\sqrt{\lambda }$ such that we obtain

(32) $$\begin{array}{c} \frac{2\sqrt{\lambda }-bp_{h}}{bp_{v}}\leq \rho \leq \frac{2-bp_{h}}{bp_{v}}. \end{array}$$

We note that the ratio *ℛ*_0*A*_^2^/*ℛ*_0*F*_^2^ ≥ 1 if and only if *ρ* ≥ (*λ* ^3/2^ + *λ* ^1/2^(*μ*_*v*_ + *μ*_*h*_ + *γ* + 1) − *bp*_*h*_)/*bp*_*v*_ = *ρ*_0_ and *ℛ*_0*A*_^2^/*ℛ*_0*F*_^2^ < 1 if and only if *ρ* < *ρ*_0_. Note also that *ρ*_0_ < (2 − *bp*_*h*_)/*bp*_*v*_ for small *λ*.

**Figure 3:**
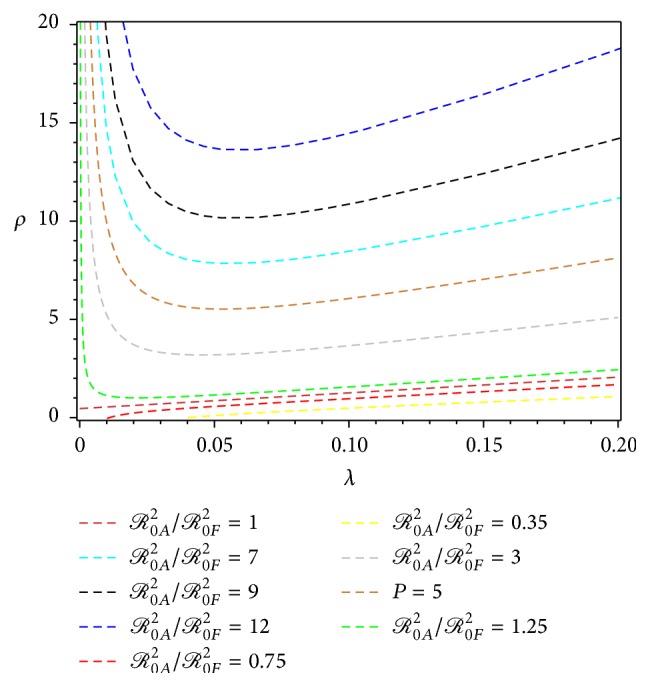
Level set *ℛ*_0*A*_^2^/*ℛ*_0*F*_^2^ in (28) for data set {*b* = 1, *p*_*h*_ = 0.01, *p*_*v*_ = 0.25, *γ* = 1/8, *μ*_*h*_ = 1/(3 × 70), *μ*_*v*_ = 1/30, *λ* ∈ [0,0.20], *ρ* ∈ [0,20]}.

**Figure 4:**
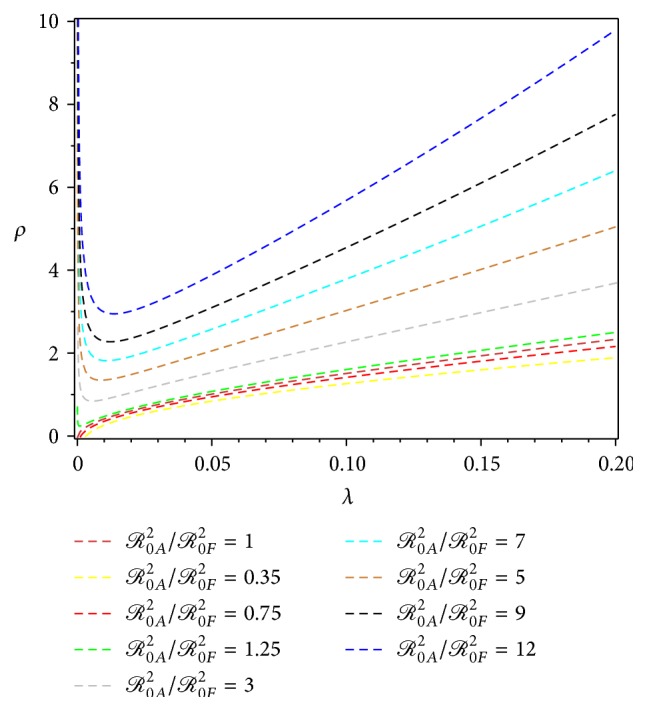
Level set *ℛ*_0*A*_^2^/*ℛ*_0*F*_^2^ in (31) for data set {*b* = 1, *p*_*h*_ = 0.01, *p*_*v*_ = 0.25, *γ* = 1/8, *μ*_*h*_ = 1/(3 × 70), *μ*_*v*_ = 1/30, *λ* ∈ [0,0.20], *ρ* ∈ [0,10]}.

Figures 3 and 4 show the level sets for some values of the ratio *ℛ*_0*A*_^2^/*ℛ*_0*F*_^2^, for both scenarios of construction of *λ*. It shows that for small *λ*, *ℛ*_0*A*_^2^/*ℛ*_0*F*_^2^ increases faster, with respect to *ρ*, in the second scenario (Figure 4) than in the first scenario (Figure 3). Summary of the construction of *ℛ*_0_ estimation for the first and the second scenario of *λ* is given in Table 1.

**Figure 5:**
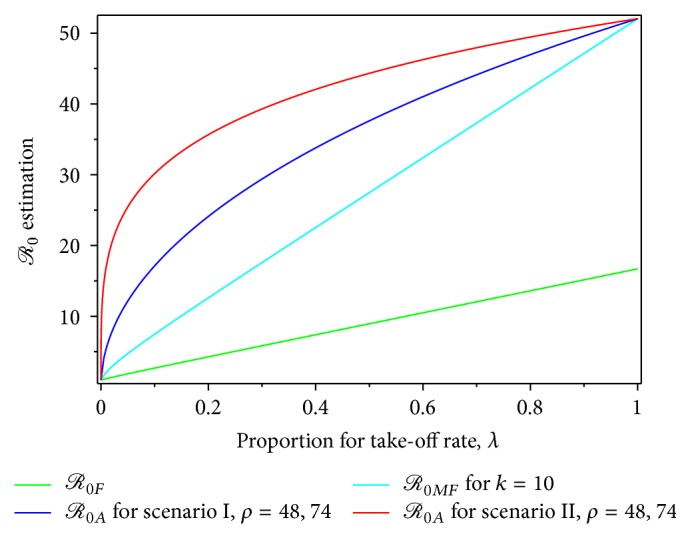
The comparison of *ℛ*_0_ estimation.

The magnitude of *ℛ*_0*A*_ in Figure 5 indicates that the second scenario is rising faster than *ℛ*_0*A*_ of the first scenario at the beginning of the growing epidemic of dengue fever, although the rate of infection is very small. This means that the presence of mosquitoes in early dengue infection directly affects the human dengue infection. Furthermore, Figure 5 shows that the second scenario of construction of *ℛ*_0_, which is summarized in Table 1, is increasing faster than the first scenario. Therefore, we obtain that the second scenario is a more realistic construction of *ℛ*_0_ than the first one at the beginning of the short period of dengue virus infection.

In the next section, we apply least square method to determine estimation of *λ*, *I*_0_, and *K*_0_ from dengue incidence data based on (27) and (30). We calculated *λ*, *I*_0_, and *K*_0_ by using least square method which differs from the estimation that has been conducted in [14]. They assume that the number of cases of dengue at *t* = 0 is equal to one, but, here, we estimate from the dengue incidence data. In the next section, we examine the dependence of the basic reproductive ratio on the take-off rate, at the beginning of the take-off period of dengue infection, for daily cases of dengue from SB Hospital in Bandung Indonesia.

## 5. Application of *ℛ*_0_ Estimation to the Real-Life Dengue Epidemic

**Figure 6:**
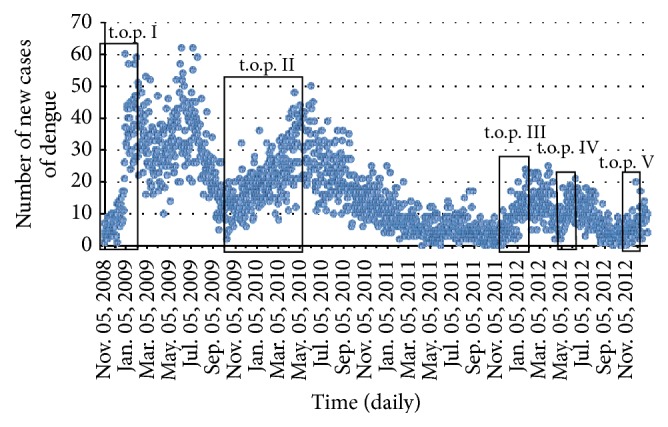
A time series of the number of daily new cases of dengue infection in the SB Hospital is from Nov. 05, 2008, to Dec. 2012. The cut off period of new cases of dengue is inside the solid box. Every t.o.p. contains i.t.o.p. of dengue infection.

This section presents the application of the method to the data of dengue incidence from SB hospital, the estimation value of the t.o.r. *λ*, the value basic reproductive ratio, and their implication. Calculation of *λ* is based on the assumption that at the beginning of the infection natural death and recovery have not yet occurred. This assumption can be taken before the eighth day of incidence. Data is divided into five take-off periods (t.o.p.) of infections, which is represented inside the solid box in Figure 6. Each t.o.p. contains an initial take-off period (i.t.o.p.) of dengue infection. Here, we define the i.t.o.p. as being within the range of the fourth and the seventh days of the dengue incidence. The correspondents of the bioepidemiological parameter from human and mosquito are given in Table 2.

Figure 6 shows the five possibilities for the t.o.p. The t.o.p. is defined by the minimum to maximum values of *r*-square (*r* ^2^). The number of days for every i.t.o.p. of incidence data depends on the value of *r* ^2^ criteria (approximately at the 4–7th day after contact with the infected mosquito). Meanwhile, the i.t.o.p. is defined by the minimum to maximum values of *r* ^2^ before the eighth day. Next we determine the rate of the i.t.o.p. of dengue infection (t.o.r.) *λ* for every t.o.p. The value of *λ* is calculated at intervals of four to seven days for the dengue of dengue infection by using two different scenarios in respect to the incidence data and the value of parameter in Table 2.

In the first scenario we calculate the values of *λ* and *I*_0_ by using least square method for (27), and, in the second scenario, we calculate the values of *λ* and *K*_0_ by using least square method for (30), for every i.t.o.p. Those values of *λ*, *I*_0_, and *K*_0_ are summarized in Table 3.

Using take-off rate values of infection *λ* in Table 3, we calculate the value of *ρ*, *ℛ*_0*F*_, *ℛ*_0*MF*_, and *ℛ*_0*A*_, which are given in Tables 4–7 as well as the intervals of $\hat{\rho }$, ${\hat{ℛ}}_{0MF}$, ${\hat{ℛ}}_{0}$, and ${\hat{ℛ}}_{0A}$ and their important notes, which are given in Tables 8–13.

The interpretation of the result in Tables 9–13, for instance, on *ℛ*_0*F*_ at t.o.p. III, is that each infective person infected 3.89 other persons via the mosquito vector at the start of the epidemic when the population was susceptible. It is similar meaning for other constructions of *ℛ*_0_ estimation. The maximum basic reproductive ratio, *ℛ*_0*F*_ found was 4.10, *ℛ*_0*MF*_ found was 5.58, and *ℛ*_0*A*_ found was 9.13, which should be compared with the maximum value which was obtained  103 in [12]. Furthermore, *ℛ*_0*F*_ is a special case for both *ℛ*_0*MF*_ and *ℛ*_0*A*_, in terms of magnitude. Furthermore, *ℛ*_0*MF*_ magnitude is a special case of *ℛ*_0*A*_ magnitude. *ℛ*_0*A*_ magnitude therefore generalized both *ℛ*_0*F*_ and *ℛ*_0*MF*_ magnitudes.  Table 4 shows that the value of *ℛ*_0*F*_ increases with an increasing *λ*. Also, *ℛ*_0*MF*_ increases with increasing *λ* and *k*. The special cases are *ℛ*_0*F*_ = *ℛ*_0*MF*_ for *k* = 1, *ℛ*_0*MF*_ < *ℛ*_0*F*_ for *k* < 1, and *ℛ*_0*F*_ < *ℛ*_0*MF*_ for *k* > 1. Thus, if we have the value of *k* from the real-life situation, then the value of *ℛ*_0*MF*_ is more realistic for estimating *ℛ*_0_ rather than *ℛ*_0*F*_.

From Tables 4 and 5, with respect to *ℛ*_0*F*_, we obtain that the average secondary case of dengue infection is at most four persons during the time period of dengue infection. Meanwhile, the value of *ℛ*_0*MF*_ depends on the value of *k*. We show that for *k* = 1, then *ℛ*_0*F*_ = *ℛ*_0*MF*_ (values in bracket). The increases in the value of *λ* and *k* cause an increase in the value of *ℛ*_0*MF*_. While the value in the square brackets in Tables 5 and 7 shows the relationship between the value of *λ* and *ρ*, so that *ℛ*_0*F*_ = *ℛ*_0*A*_. We also found that an increase in the value of *λ*, for a particular value of *ρ*, does not always cause an increase in the value of *ℛ*_0*A*_. Particular information for the four t.o.p. in Tables 6 and 7 shows that, to obtain the four secondary infection cases from one primary infection, the number of mosquitoes should be at least twice as large as the human population, whereas in Tables 4 and 5 we do not obtain information about the ratio of the number of mosquitoes and humans. Therefore, *ℛ*_0*A*_ provides more complete information than *ℛ*_0*F*_ and *ℛ*_0*MF*_.

Next, From Tables 6 and 7, there is the value of *ρ* such that *ℛ*_0*F*_ = *ℛ*_0*A*_ for every dengue infection i.t.o.p. (see the value in brackets of Tables 6 and 7). For *k* = 1, there are a certain pair of *ρ* and *λ* for every i.t.o.p., such that *ℛ*_0*A*_ = *ℛ*_0*MF*_ = *ℛ*_0*F*_ (Table 4). For the first scenario, if *ρ* is relatively small then the value of *ℛ*_0*A*_ is inversely proportional to the value of *λ*. For instance, if *ρ* = 0.67 (see t.o.p. II, Table 6) then *ℛ*_0*A*_ increases or decreases with an increasing *λ*. The magnitude relation between *λ* and *ℛ*_0_ is not the case in other values of *ρ*. Also, we obtained that *ℛ*_0*F*_ is a special case of *ℛ*_0*A*_ for a certain pair of *ρ* and *λ*. Thus, if we have the value of *ρ* from the real-life situation then the value of *ℛ*_0*A*_ is more realistic than *ℛ*_0*F*_ when estimating *ℛ*_0_ from the real-life dengue epidemic.

Next, in the first scenario, the value of *ℛ*_0*F*_ is not only contained in ${\hat{ℛ}}_{0MF}$ and ${\hat{ℛ}}_{0A}$ but also contained within ${\hat{ℛ}}_{0}$. Also, the value of *ℛ*_0*F*_ belongs to the intersection of ${\hat{ℛ}}_{0MF}$ and ${\hat{ℛ}}_{0A}$. This is also the case in the first scenario, where *ℛ*_0*F*_ have good precision but not as good a predictor when compared with ${\hat{ℛ}}_{0MF}$ and ${\hat{ℛ}}_{0A}$. Whilst ${\hat{ℛ}}_{0MF}$ and ${\hat{ℛ}}_{0A}$ are good predictors, they do not have a good precision (see Tables 7–13). In the second scenario, the value of ${\hat{ℛ}}_{0F}$ is contained within the range of ${\hat{ℛ}}_{0MF}$ and ${\hat{ℛ}}_{0}$ values but not contained in ${\hat{ℛ}}_{0A}$ range. Furthermore, the value of *ℛ*_0*F*_ is lower than range's lower end of values of ${\hat{ℛ}}_{0A}$ (see Tables 10 [Table tab11] [Table tab12]–13). Meanwhile, it is not enough just to pay attention to ${\hat{ℛ}}_{0}$ value of the model in (1). Therefore, from the aspect of the efficiency and effectiveness of dengue control, the government should pay attention to the basic reproductive ratio estimation of *ℛ*_0*A*_ that is reported here, in order to improve the quality control of the spread of dengue fever.

The important notes in Tables 10–13 show that *ℛ*_0*F*_ value (see Table 4 or Tables 5–7) is included in the intervals ${\hat{ℛ}}_{0MF}$, ${\hat{ℛ}}_{0A}$, and ${\hat{ℛ}}_{0}$ for the first scenario. It means that the value of *ℛ*_0*F*_ can still be used as a standard for the control of dengue virus infection. However, to obtain the maximum value of *ℛ*_0*A*_ = 2 − 12, then the number of mosquitoes would be 2–8 times greater than the number of people, with the take-off rate of 0.121; see Table 11. Therefore, the use of value of *ρ*, *λ*, and *ℛ*_0*A*_ as a standard value for the control of a dengue endemicity is more realistic than the others.

**Figure 7:**
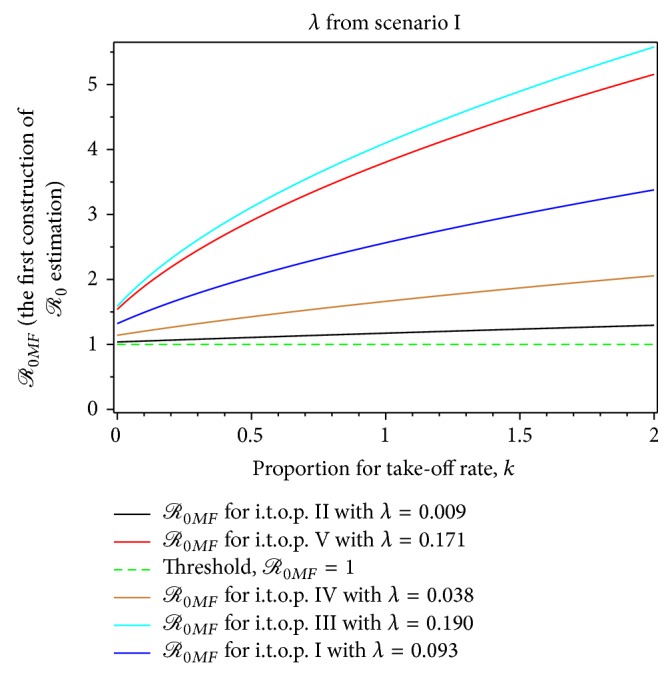
The dynamic of *ℛ*_0*MF*_ for the dengue outbreak at the beginning of the t.o.p. as a function of the infected mosquito per human index for *λ* from the first scenario.

**Figure 8:**
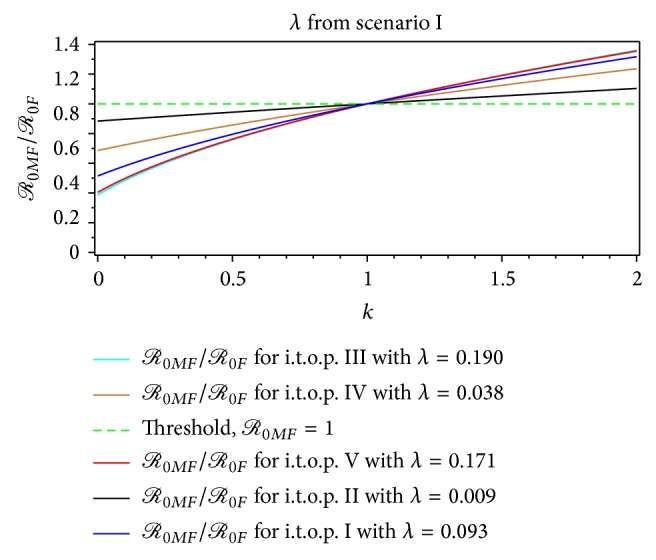
The ratio of *ℛ*_0*MF*_/*ℛ*_0*F*_ for the dengue outbreak at the beginning of the t.o.p. as a function of the ratio of the rate of the infected mosquito per human index *k* for *λ* from the first scenario.

**Figure 9:**
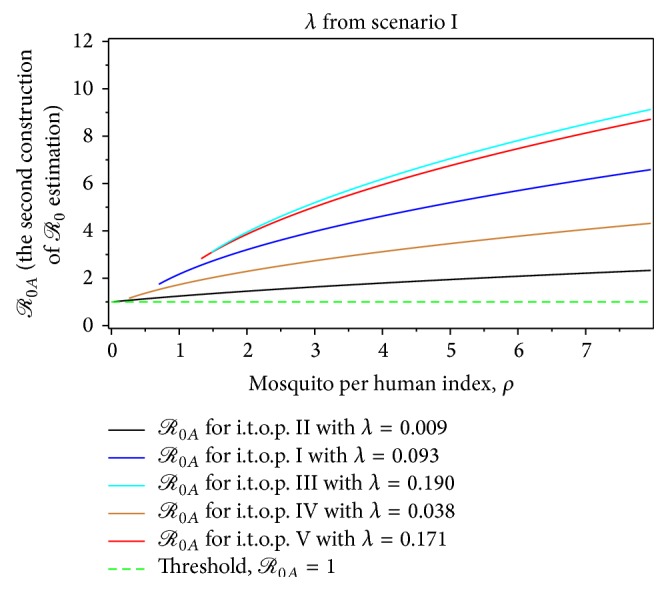
The dynamic of *ℛ*_0*A*_ for the dengue outbreak at the beginning of the t.o.p. as a function of the mosquito per person index for *λ* from the first scenario.

**Figure 10:**
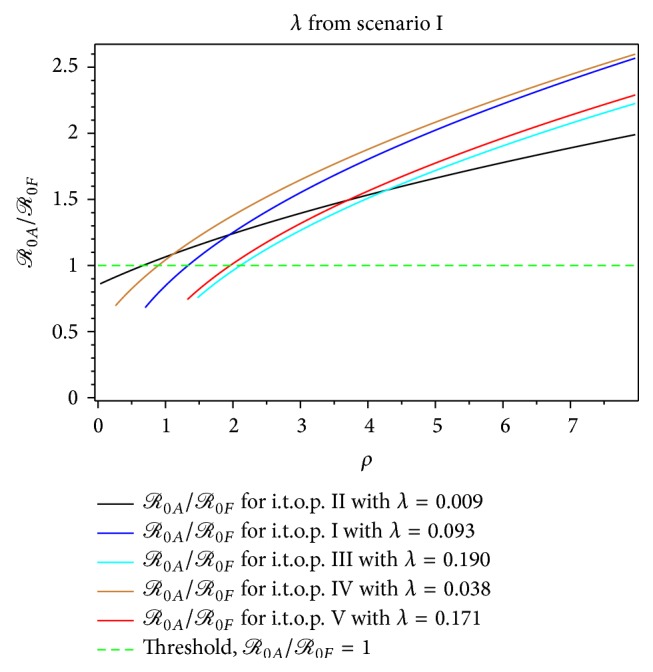
The ratio of *ℛ*_0*A*_/*ℛ*_0*F*_ for the dengue outbreak at the beginning of the t.o.p. as a function of the infected mosquito per human index *ρ* for *λ* from the first scenario.

**Figure 11:**
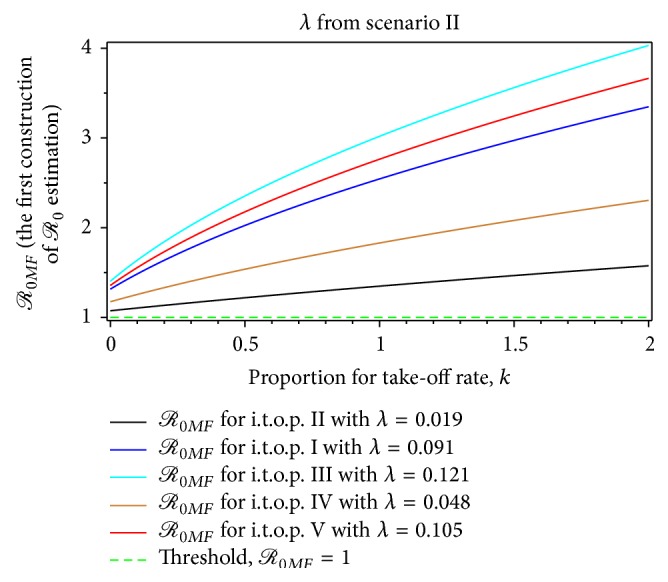
The dynamic of *ℛ*_0*MF*_ for the dengue outbreak at the beginning of the t.o.p. as a function of the infected mosquito per human index for *λ* from the first Scenario.

**Figure 12 fig12:**
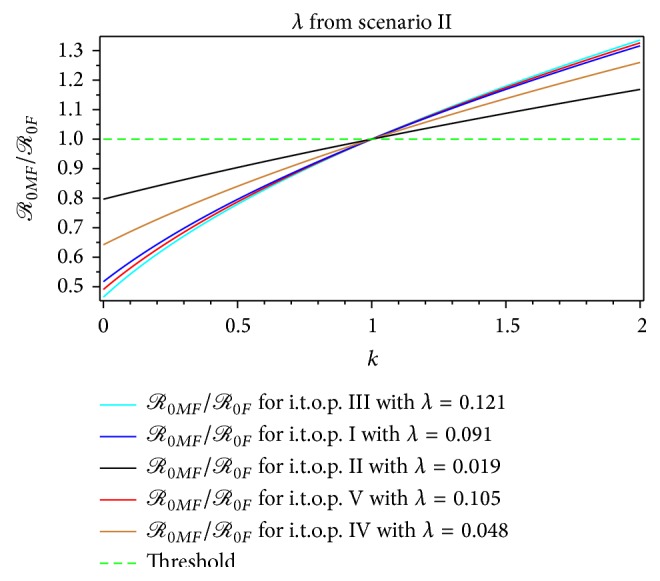
The ratio of *ℛ*_0*MF*_/*ℛ*_0*F*_ for the dengue outbreak at the beginning of the t.o.p. as a function of ratio of the rate of the infected mosquito per human index *k* for *λ* from the second scenario.

**Figure 13 fig13:**
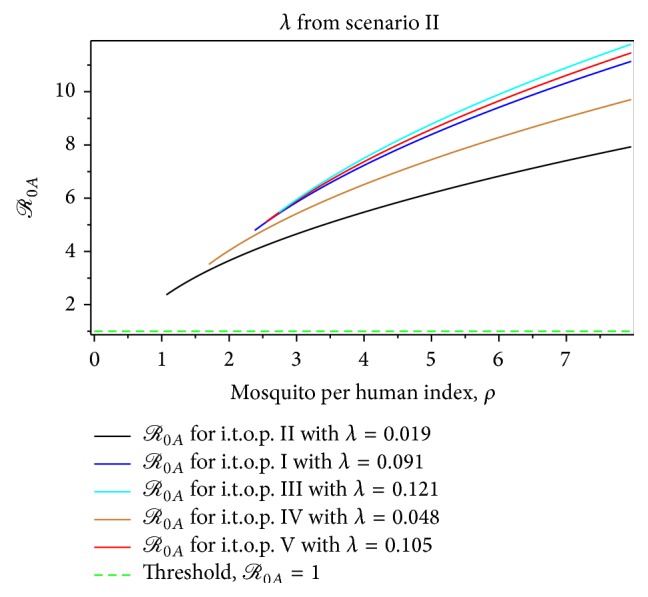
The dynamic of *ℛ*_0*A*_ for the dengue outbreak at the beginning of the t.o.p. as a function of the mosquito per person index for *λ* from the second scenario.

**Figure 14:**
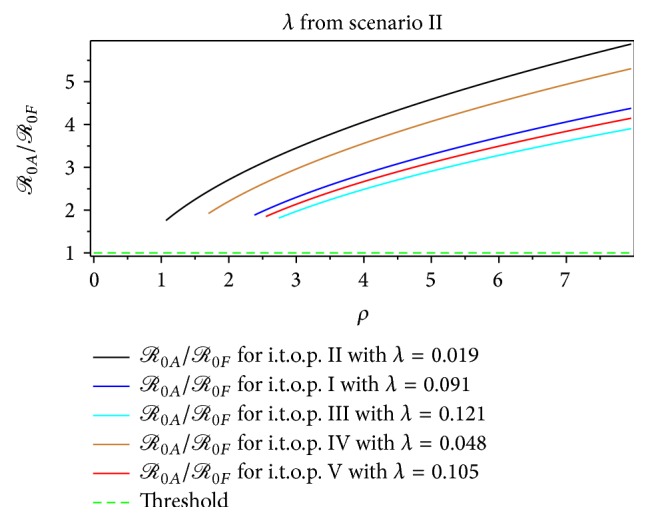
The ratio of *ℛ*_0*A*_/*ℛ*_0*F*_ for the dengue outbreak at the beginning of the t.o.p. as a function of the infected mosquito per human index *ρ* for *λ* from the second scenario.

From Figures 7–14, not only do we obtain the value of the basic reproductive ratio estimation of the *ℛ*_0*A*_ which is greater than one but also we do obtain the value which is less than one; for instance, from the first scenario at *λ* = 0.190 and *ρ* = 0.67 we have *ℛ*_0*A*_ = 0.67 < 1 (see Table 7). Also, we can determine the intersection range of a basic reproductive ratio from a different construction of value of *λ*. Both proposed constructions of *ℛ*_0_ are the same when using assumption in [12] for these particular cases. But increasing the number of secondary cases for every t.o.p. will depend on *k* for the first construction and depend on *ρ* for the second construction. These results indicate that the greater the number of mosquitoes means the higher the number of new cases of dengue and the shorter the period of the i.t.o.p. of dengue infection. Furthermore, *ℛ*_0*F*_ estimates of *ℛ*_0_ are too low when compared to *ℛ*_0*A*_. This is very dangerous in application, since the intervention to control dengue that results from *ℛ*_0*F*_ estimation will not be able to stop the disease once it exists. Therefore, prevention of dengue incidences is more appropriate if it is done early, and exactly how early is dependent on *ℛ*_0*A*_ estimation.

We used real dengue epidemic data from a dengue epidemic, between the dates Nov. 25, 2008, and 2012 from SB Hospital. This includes the estimation of the basic reproductive ratio at the beginning of t.o.p. of dengue infection by using *ℛ*_0*F*_, *ℛ*_0*MF*_, and *ℛ*_0*A*_ for *ℛ*_0_ estimation. *ℛ*_0_ magnitude estimation under the same assumption was conducted in [12], resulting in *ℛ*_0*F*_. We propose *ℛ*_0*MF*_ and *ℛ*_0*A*_ for comparing with *ℛ*_0*F*_ for *ℛ*_0_ estimation from dengue incidence data from SB Hospital. As a results, we get *ℛ*_0*MF*_ > 1.59 from the first scenario (Table 4, Figures 7 and 9) and *ℛ*_0*MF*_ > 1.40 from the second scenario, respectively (Table 6, Figures 7 and 9). From the first scenario, we obtained the the basic reproductive ratio was lowest in the t.o.p. II [1.01,2.33) and largest in the t.o.p. III [3.10,9.13) (Table 10, Figures 8 and 10). Furthermore, from the second scenario, the basic reproductive ratio was lowest in the t.o.p. II [2 .37,7.93) and largest in the t.o.p. III [5.48,11.78) (Table 11, Figures 8 and 10). We believe that *ℛ*_0*MF*_ and *ℛ*_0*A*_ are a good *ℛ*_0_ estimation at the beginning of the take-off period of infection. Thus, our results can be considered in the control of a dengue fever epidemic, in the dengue endemic regions of Bandung, Indonesia. But the effects of dengue incidence data were obtained from SB Hospital, Bandung, to the value of *ℛ*_0_ estimation, and describe the transmission of dengue fever, because only the dengue incidence data of the same* serotype* were considered.

Some of the results of the basic reproductive ratio estimation for dengue fever in compartmental model were reported in several publications. Favier et al. in [12] estimated a basic reproductive ratio, which ranged widely from 2.0 to 103 for dengue epidemics occurring during the years 1996 to 2003 in 9 Brazilian regions. The upper bound of their range, in estimating the basic reproductive ratio, is significantly higher than the upper bound estimate reported here. The lower bound of their range estimate, of basic reproductive ratio, is relatively smaller than the lower bound estimate reported here for some take-off period but the others are relatively higher than the lower bound estimate reported here (Tables 4 and 6). Chowell et al. in [11] estimated the basic reproductive ratio, by using the relationship between the basic reproductive ratio and the growth rate in the early phase of dengue fever transmission in Toceman, Mexico. Their mean estimate of the basic reproductive ratio is 4.22, which is significantly higher than the mean estimate reported here for every take-off period which is obtained by *ℛ*_0*F*_. The weekly number of dengue cases in Peru for the period 1994–2006 was analyzed in [21] which obtained that the basic reproductive ratio had a median 1.76 (0.83–4.46), where these values inside the value of *ℛ*_0_ are reported here; see Table 7. During the 2000 epidemic, occurring in 12 cities of the state of Sao Paulo, Brazil, [4] estimated that the basic reproductive ratio was in the range [3.6,12.9]. More recently [14] estimated the reproductive ratio by using the relationship between the basic reproductive ratio and the force of infection of dengue fever transmission in Salvador from periods 1996 to 1997, and 2002. They obtain an estimation of *ℛ*_0_ = 2.85(2.77,3.70) in the periods 1996 to 1997, and *ℛ*_0_ = 2.65(2.50,3.30) for 2002. The newest report of *ℛ*_0_ estimation is in [22] for dengue epidemics in 2002 dengue outbreak in Easter Island, Chile. Their mean of the basic reproductive ratio *ℛ*_0_ at 27.2 is significantly higher than every value of *ℛ*_0_ estimation reported here for five initial take-off periods of dengue infection from two scenarios for estimating of *λ*. Our estimation results, for the basic reproductive ratio by using *ℛ*_0*F*_ range widely, are from 1.17 to 3.89 for the first scenario and from 1.35 to 3.02 for the second scenario which belongs to the range of the first scenario. Our *ℛ*_0*MF*_ estimation accommodates for the difference of the take-off rate *λ* between mosquito and human. If there are no differences in the take-off rate then [12] estimates are exactly the same as ours. Otherwise *ℛ*_0*F*_ could overestimate or underestimate depending on the value of *k*. Tables 4 and 6 show that *ℛ*_0*A*_ accommodates differences in *ρ*. *ℛ*_0*F*_ is exactly the same as our second estimates for a certain pair of *ρ* and *λ*. Otherwise *ℛ*_0*F*_ could overestimate or underestimate depending on the value of *ρ*, and so forth. Therefore, our *ℛ*_0*MF*_ and *ℛ*_0*A*_ constructions are more realistic for estimating the value of the basic reproductive ratio, than the *ℛ*_0*F*_.

In the first scenario, the control of dengue fever, which is based on the standard value *ℛ*_0*F*_, is still realistic. Furthermore, better control of dengue fever, when based on the maximum value of *ℛ*_0*MF*_ or maximum value of *ℛ*_0*A*_, is shown in Table 6 and Figures 7–10. While for the second scenario, which is shown by Table 6 and Figures 11 [Fig fig12] [Fig fig13]–14, it is better to choose one value from the interval as a standard value used to control dengue fever. Therefore, the second construction is better choice, for dengue control strategies in Bandung, Indonesia. Nevertheless, the estimation of *ℛ*_0_ has limitations, because it is only done from the fourth day until the seventh day, so the effect of the increase in the incidence of dengue *ℛ*_0_ is no longer considered. Furthermore, the dengue incidence data used, from SB Hospital, do not distinguish between the latent population and the dengue infected human population, so the estimate of *ℛ*_0_ is limited by the data characteristics. In reality, nobody can distinguish between the latent population and the dengue infected population. Furthermore the latent population has a short incubation time period.

## 6. Conclusion

We have conducted two different constructions of basic reproductive ratio estimations based on incidence data. These constructions modify *ℛ*_0*F*_ model by allowing the take-off rates of human and mosquito to vary. In the first construction, we assumed that the take-off rate of an infected mosquito is proportional to the take-off rate of a human. This construction leads to a new form of modified basic reproductive ratio estimation for *ℛ*_0*MF*_ was a function of the take-off rate of a human (*λ*) and the take-off rate of a mosquito (*kλ*). A sensitivity analysis indicated that a small variation of a mosquito take-off rate could affect significant change in the basic reproductive ratio. In the second construction we assumed that, during the take-off period, the initial growth of infected humans is slightly affected by the growth of infected mosquitoes, and vice versa. This construction leads to a one-parameter coupling of the initial growth of infected human and mosquitoes. The modified basic reproductive ratio estimation for *ℛ*_0*A*_ resulted from this construction, and it depends on the mosquitoes per person index *ρ*. This ratio increases for small take-off rate and decreases for larger take-off rate. A numerical analysis of *ℛ*_0*MF*_/*ℛ*_0*F*_ and *ℛ*_0*A*_/*ℛ*_0*F*_ ratios shows variation in a relatively large range of values for *ℛ*_0*F*_, in respect to (*λ*, *k*) and (*λ*, *ρ*) for the first and second scenario for determined *λ*, respectively.

## Figures and Tables

**Table 1:** Summary of the construction of *ℛ*_0_ and *λ* estimation for the first scenario.

The first scenario for *λ*	*ℛ*_0_ estimation
Based on [12] assumption	${ℛ}_{0F}^{2}=\frac{\lambda^{2}}{\mu_{v}\left(\mu_{h}+\gamma \right)}+\frac{\left(\mu_{v}+\mu_{h}+\gamma \right)\lambda }{\mu_{v}\left(\mu_{h}+\gamma \right)}+1$
The first construction	${ℛ}_{0MF}^{2}=\frac{k\lambda^{2}}{\mu_{v}\left(\mu_{h}+\gamma \right)}+\frac{\left(\mu_{v}+k\left(\mu_{h}+\gamma \right)\right)\lambda }{\mu_{v}\left(\mu_{h}+\gamma \right)}+1$
The second construction	${ℛ}_{0A}^{2}=-\frac{\lambda^{2}}{\mu_{v}\left(\gamma +\mu_{h}\right)}+\frac{b\left(p_{h}+p_{v}\rho \right)}{\mu_{v}\left(\gamma +\mu_{h}\right)}\lambda +1$
The t.o.r. *λ* equation	*I*(*t*) = *λI*_0_ *t* + *I*_0_

The second scenario for *λ*	*ℛ*_0_ estimation

Based on [12] assumption	${ℛ}_{0F}^{2}=\frac{\lambda^{2}}{\mu_{v}\left(\mu_{h}+\gamma \right)}+\frac{\left(\mu_{v}+\mu_{h}+\gamma \right)\lambda }{\mu_{v}\left(\mu_{h}+\gamma \right)}+1$
The first construction	${ℛ}_{0MF}^{2}=\frac{k\lambda^{2}}{\mu_{v}\left(\mu_{h}+\gamma \right)}+\frac{\left(\mu_{v}+k\left(\mu_{h}+\gamma \right)\right)\lambda }{\mu_{v}\left(\mu_{h}+\gamma \right)}+1$
The second construction	${ℛ}_{0A}^{2}=-\frac{\lambda }{\mu_{v}\left(\gamma +\mu_{h}\right)}+\frac{b\left(p_{h}+p_{v}\rho \right)}{\mu_{v}\left(\gamma +\mu_{h}\right)}\sqrt{\lambda }+1$
The t.o.r. *λ* equation	*I*(*t*) = *λK*(*t*)+(1 − *λ*)*K*_0_

**Table 2:** Bioepidemiological data of human and mosquito.

Symbol	Definition	Value
*b*	Biting rate (day^−1^)	1
*p*_*h*_	Effective contact rate to human	0.01
*p*_*v*_	Effective contact rate to mosquito	0.25
*μ*_*h*_	Human mortality rate (day^−1^)	$\frac{1}{365\times 70}$
*μ*_*v*_	Mosquito mortality rate (day^−1^)	$\frac{1}{30}$
*γ*	Recovering rate of human (day^−1^)	$\frac{1}{8}$
*ρ*	Mosquito per human index	0–7.96
*k*	Proportion of mosquito per human index	0–2

**Table 3:** The value of *λ* and *I*_0_ from the first scenario and the value of *λ* and *K*_0_ for the second scenario, respectively.

Take-off period	Initial take-off period	The values of *λ* and *I*_0_ for the first scenario	The values of *λ* and *K*_0_ for the second scenario
I: 103 days	I: 5 days	*λ* = 0.093, *I*_0_ = 5.4	*λ* = 0.091, *K*_0_ = 5.22
II: 187 days	II: 6 days	*λ* = 0.009, *I*_0_ = 11.53	*λ* = 0.019, *K*_0_ = 11.17
III: 13 days	III: 6 days	*λ* = 0.190, *I*_0_ = 3.62	*λ* = 0.121, *K*_0_ = 3.78
IV: 17 days	IV: 5 days	*λ* = 0.038, *I*_0_ = 16	*λ* = 0.048, *K*_0_ = 15.54
V: 85 days	V: 7 days	*λ* = 0.171, *I*_0_ = 2.93	*λ* = 0.105, *K*_0_ = 3.1

**Table 4:** The values of *ℛ*_0*F*_ for a given value of *λ* at the i.t.o.p. and the corresponding values of *ℛ*_0*MF*_ for different values of *k* for the first scenario.

The *n* days at the i.t.o.p.	*λ*	*ℛ*_0*F*_	*ℛ*_0*MF*_	*ℛ*_0*MF*_	*ℛ*_0*MF*_	*ℛ*_0*MF*_	*ℛ*_0*MF*_
			*k* = 0	*k* = 0.75	*k* = 1	*k* = 1.25	*k* = 2
II: 7 days	0.009	[1.17]	1.04	1.14	[1.17]	1.20	1.29
IV: 5 days	0.038	[1.66]	1.14	1.55	[1.66]	1.77	2.06
I: 5 days	0.093	[2.56]	1.32	2.32	[2.56]	2.79	3.38
V: 7 days	0.171	[3.80]	1.54	3.38	[3.80]	4.18	5.16
III: 6 days	0.190	[3.89]	1.59	3.64	[3.89]	4.52	5.58

**Table 5:** The values of *ℛ*_0*F*_ for a given value of *λ* at the i.t.o.p. and the corresponding values of *ℛ*_0*MF*_ for different values of *k* for the second scenario.

The *n* days at the i.t.o.p.	*λ*	*ℛ*_0*F*_	*ℛ*_0*MF*_	*ℛ*_0*MF*_	*ℛ*_0*MF*_	*ℛ*_0*MF*_	*ℛ*_0*MF*_
			*k* = 0	*k* = 0.75	*k* = 1	*k* = 1.25	*k* = 2
II: 7 days	0.019	[1.35]	1.07	1.29	[1.35]	1.41	1.58
IV: 5 days	0.048	[1.83]	1.17	1.69	[1.83]	1.96	2.30
I: 5 days	0.091	[2.54]	1.32	2.30	[2.54]	2.77	3.35
V: 7 days	0.105	[2.76]	1.36	2.49	[2.76]	3.01	3.66
III: 6 days	0.121	[3.02]	1.40	2.71	[3.02]	3.30	4.03

**Table 6:** The values of *ℛ*_0*F*_ for every value of *λ* at each initial t.o.p. and the values of *ℛ*_0*A*_ for every change in the value of *λ* and *ρ* for the first scenario for calculation of *λ*.

The *n* days at the i.t.o.p.	*λ*	*ℛ*_0*F*_	*ℛ*_0*A*_	*ℛ*_0*A*_	*ℛ*_0*A*_	*ℛ*_0*A*_	*ℛ*_0*A*_
			*ρ* = 0.67	*ρ* = 0.89	*ρ* = 1.33	*ρ* = 1.96	*ρ* = 2.11
II: 7 days	0.009	[1.17]	[1.17]	1.22	1.32	1.45	1.48
IV: 5 days	0.038	[1.66]	1.50	[1.66]	1.94	2.27	2.34
I: 5 days	0.093	[2.56]	1.70	2.03	[2.56]	3.17	3.30
V: 7 days	0.171	[3.80]	1.12	1.89	2.84	[3.80]	4.00
III: 6 days	0.190	[4.10]	0.67	1.73	2.83	3.89	[4.10]

**Table 7:** The values of *ℛ*_0*F*_ for every value of *λ* at each initial t.o.p. and the values of *ℛ*_0*A*_ for every change in the value of *λ* and *ρ* for the second scenario for calculating of *λ*.

The *n* days at the i.t.o.p.	*λ*	*ℛ*_0*F*_	*ℛ*_0*A*_	*ℛ*_0*A*_	*ℛ*_0*A*_	*ℛ*_0*A*_	*ℛ*_0*A*_
			*ρ* = 0.61	*ρ* = 1.01	*ρ* = 1.47	*ρ* = 1.60	*ρ* = 1.74
II: 7 days	0.019	[1.35]	[1.35]	2.27	2.99	3.16	3.34
IV: 5 days	0.048	[1.83]	—	[1.83]	3.06	3.32	3.59
I: 5 days	0.091	[2.54]	—	—	[2.54]	2.96	3.37
V: 7 days	0.105	[2.76]	—	—	2.27	[2.76]	3.23
III: 6 days	0.121	[3.02]	—	—	1.87	2.48	[3.02]

**Table 8:** Summary of the ${\hat{ℛ}}_{0}$ and ${\hat{ℛ}}_{0MF}$ for the first scenario of the value of *λ* and *ρ* in (29).

The *n* days at the i.t.o.p.	*λ*	$\hat{\rho }$	${\hat{ℛ}}_{0MF}$	${\hat{ℛ}}_{0}$	Important notes
		from (29)	for *k* ∈ (0,2]		
II: 7 days	0.009	(0.034,7.96)	(1.04,1.29]	(0.02,4.77)	${ℛ}_{0F}\in {\hat{ℛ}}_{0MF}\subset {\hat{ℛ}}_{0}$
IV: 5 days	0.038	(0.26,7.96)	(1.14,2.06]	(0.16,4.77)	${ℛ}_{0F}\in {\hat{ℛ}}_{0MF}\subset {\hat{ℛ}}_{0}$
I: 5 days	0.093	(0.701,7.96)	(1.32,3.38]	(0.42,4.77)	${ℛ}_{0F}\in {\hat{ℛ}}_{0MF}\subset {\hat{ℛ}}_{0}$
V: 7 days	0.171	(1.33,7.96)	(1.54,5.16]	(0.80,4.77)	${ℛ}_{0F}\in {\hat{ℛ}}_{0}\cap {\hat{ℛ}}_{0MF}$
III: 6 days	0.190	(1.48,7.96)	(1.59,5.58]	(0.89,4.77)	${ℛ}_{0F}\in {\hat{ℛ}}_{0}\cap {\hat{ℛ}}_{0MF}$

*Note*. The value of *ℛ*_0*F*_ from Table 6.

**Table 9:** Summary of the ${\hat{ℛ}}_{0}$ and ${\hat{ℛ}}_{0MF}$ for the second scenario of the value of *λ* and *ρ* in (32).

The *n* days at the i.t.o.p.	*λ*	$\hat{\rho }$	${\hat{ℛ}}_{0}$	${\hat{ℛ}}_{0MF}$	Important notes
		from (32)		for *k* ∈ (0,2]	
II: 7 days	0.019	(1.07,7.96)	(0.64,4.77)	(1.07,1.58)	${ℛ}_{0F}\in {\hat{ℛ}}_{0MF}\subset {\hat{ℛ}}_{0}$
IV: 5 days	0.048	(1.71,7.96)	(1.02,4.77)	(1.17,2.30)	${ℛ}_{0F}\in {\hat{ℛ}}_{0MF}\subset {\hat{ℛ}}_{0}$
I: 5 days	0.091	(2.37,7.96)	(1.43,4.77)	(1.32,3.35)	${ℛ}_{0F}\in {\hat{ℛ}}_{0}\cap {\hat{ℛ}}_{0MF}$
V: 7 days	0.105	(2.55,7.96)	(1.53,4.77)	(1.36,3.66)	${ℛ}_{0F}\in {\hat{ℛ}}_{0}\cap {\hat{ℛ}}_{0MF}$
III: 6 days	0.121	(2.74,7.96)	(1.65,4.77)	(1.40,4.03)	${ℛ}_{0F}\in {\hat{ℛ}}_{0}\cap {\hat{ℛ}}_{0MF}$

*Note*. The value of *ℛ*_0*F*_ from Table 7.

**Table 10:** Summary of the *ℛ*_0_ and *ℛ*_0*A*_ for the first scenario of the value of *λ* and *ρ* in (29).

The *n* days at the i.t.o.p.	*λ*	$\hat{\rho }$	${\hat{ℛ}}_{0}$	${\hat{ℛ}}_{0A}$	Important notes
		from (29)			
II: 7 days	0.009	(0.034,7.96)	(0.02,4.77)	(1.01,2.33)	${ℛ}_{0F}\in {\hat{ℛ}}_{0A}\subset {\hat{ℛ}}_{0}$
IV: 5 days	0.038	(0.26,7.96)	(0.16,4.77)	(1.16,4.32)	${ℛ}_{0F}\in {\hat{ℛ}}_{0A}\subset {\hat{ℛ}}_{0}$
I: 5 days	0.093	(0.701,7.96)	(0.42,4.77)	(1.75,6.59)	${ℛ}_{0F}\in {\hat{ℛ}}_{0}\cap {\hat{ℛ}}_{0A}$
V: 7 days	0.171	(1.33,7.96)	(0.80,4.77)	(2.83,8.71)	${ℛ}_{0F}\in {\hat{ℛ}}_{0}\cap {\hat{ℛ}}_{0A}$
III: 6 days	0.190	(1.48,7.96)	(0.89,4.77)	(3.10,9.13)	${ℛ}_{0F}\in {\hat{ℛ}}_{0}\cap {\hat{ℛ}}_{0A}$

*Note*. The value of *ℛ*_0*F*_ from Table 6.

**Table 11 tab11:** Summary of the ${\hat{ℛ}}_{0}$ and ${\hat{ℛ}}_{0A}$ for the second scenario of the value of *λ* and *ρ* in (32).

The *n* days at the i.t.o.p.	*λ*	$\hat{\rho }$	${\hat{ℛ}}_{0}$	${\hat{ℛ}}_{0A}$	Important notes
		from (32)			
II: 7 days	0.019	(1.07,7.96)	(0.64,4.77)	(2.37,7.93)	${ℛ}_{0F}\in {\hat{ℛ}}_{0}\setminus {\hat{ℛ}}_{0A}$
IV: 5 days	0.048	(1.704,7.96)	(1.02,4.77)	(3.52,9.70)	${ℛ}_{0F}\in {\hat{ℛ}}_{0}\setminus {\hat{ℛ}}_{0A}$
I: 5 days	0.091	(2.37,7.96)	(1.43,4.77)	(4.79,11.14)	${ℛ}_{0F}\in {\hat{ℛ}}_{0}\setminus {\hat{ℛ}}_{0A}$
V: 7 days	0.105	(2.55,7.96)	(1.53,4.77)	(5.12,11.46)	${ℛ}_{0F}\in {\hat{ℛ}}_{0}\setminus {\hat{ℛ}}_{0A}$
III: 6 days	0.121	(2.74,7.96)	(1.65,4.77)	(5.48,11.78)	${ℛ}_{0F}\in {\hat{ℛ}}_{0}\setminus {\hat{ℛ}}_{0A}$

*Note*. The value of *ℛ*_0*F*_ from Table 7.

**Table 12 tab12:** The value of *ℛ*_0*F*_ is related to ${\hat{ℛ}}_{0MF}$, ${\hat{ℛ}}_{0}$, and ${\hat{ℛ}}_{0A}$ for the first scenario.

The *n* days at the i.t.o.p.	*λ*	*ℛ*_0*F*_	${\hat{ℛ}}_{0MF}$	Important notes
			for *k* ∈ (0,2]	
II: 7 days	0.009	1.17	(1.04,1.29]	${ℛ}_{0F}\in {\hat{ℛ}}_{0MF}\cap {\hat{ℛ}}_{0}\cap {\hat{ℛ}}_{0A}$
IV: 5 days	0.038	1.66	(1.14,2.06]	${ℛ}_{0F}\in {\hat{ℛ}}_{0MF}\cap {\hat{ℛ}}_{0}\cap {\hat{ℛ}}_{0A}$
I: 5 days	0.093	2.56	(1.32,3.38]	${ℛ}_{0F}\in {\hat{ℛ}}_{0MF}\cap {\hat{ℛ}}_{0}\cap {\hat{ℛ}}_{0A}$
V: 7 days	0.171	3.80	(1.54,5.16]	${ℛ}_{0F}\in {\hat{ℛ}}_{0MF}\cap {\hat{ℛ}}_{0}\cap {\hat{ℛ}}_{0A}$
III: 6 days	0.190	3.89	(1.59,5.58]	${ℛ}_{0F}\in {\hat{ℛ}}_{0MF}\cap {\hat{ℛ}}_{0}\cap {\hat{ℛ}}_{0A}$

*Note*. ${\hat{ℛ}}_{0A}$ and ${\hat{ℛ}}_{0}$ are from Table 10.

**Table 13:** The value of *ℛ*_0*F*_ is related to ${\hat{ℛ}}_{0MF}$, ${\hat{ℛ}}_{0}$, and ${\hat{ℛ}}_{0A}$ for the second scenario.

The *n* days at the i.t.o.p.	*λ*	*ℛ*_0*F*_	${\hat{ℛ}}_{0MF}$	Important notes
			for *k* ∈ (0,2]	
II: 7 days	0.019	1.35	(1.07,1.58]	${ℛ}_{0F}\in \left({\hat{ℛ}}_{0MF}\cap {\hat{ℛ}}_{0}\right)\setminus {\hat{ℛ}}_{0A}$
IV: 5 days	0.048	1.83	(1.17,2.30]	${ℛ}_{0F}\in ({\hat{ℛ}}_{0MF}\cap {\hat{ℛ}}_{0})\setminus {\hat{ℛ}}_{0A}$
I: 5 days	0.091	2.54	(1.32,3.35]	${ℛ}_{0F}\in ({\hat{ℛ}}_{0MF}\cap {\hat{ℛ}}_{0})\setminus {\hat{ℛ}}_{0A}$
V: 7 days	0.105	2.76	(1.36,3.66]	${ℛ}_{0F}\in ({\hat{ℛ}}_{0MF}\cap {\hat{ℛ}}_{0})\setminus {\hat{ℛ}}_{0A}$
III: 6 days	0.121	3.02	(1.40,4.03]	${ℛ}_{0F}\in ({\hat{ℛ}}_{0MF}\cap {\hat{ℛ}}_{0})\setminus {\hat{ℛ}}_{0A}$

${\hat{ℛ}}_{0A}$ and ${\hat{ℛ}}_{0}$ are from Table 11.
